# Supplementary material for: Low-abundance populations distinguish microbiome performance in plant cell wall deconstruction
Source: Microbiome. 2022 Oct 25;10:183. doi: 10.1186/s40168-022-01377-x (PMC9594917; doi:10.1186/s40168-022-01377-x)

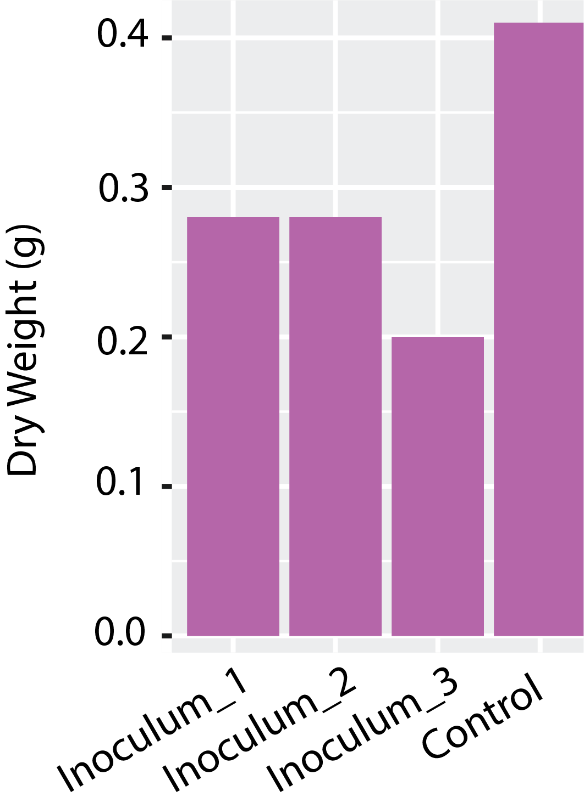


Supplementary Fig. 1. Residual dry weight biomass after a 56-day incubation. The figure shows that three incubations diverge in terms of biomass degradation.


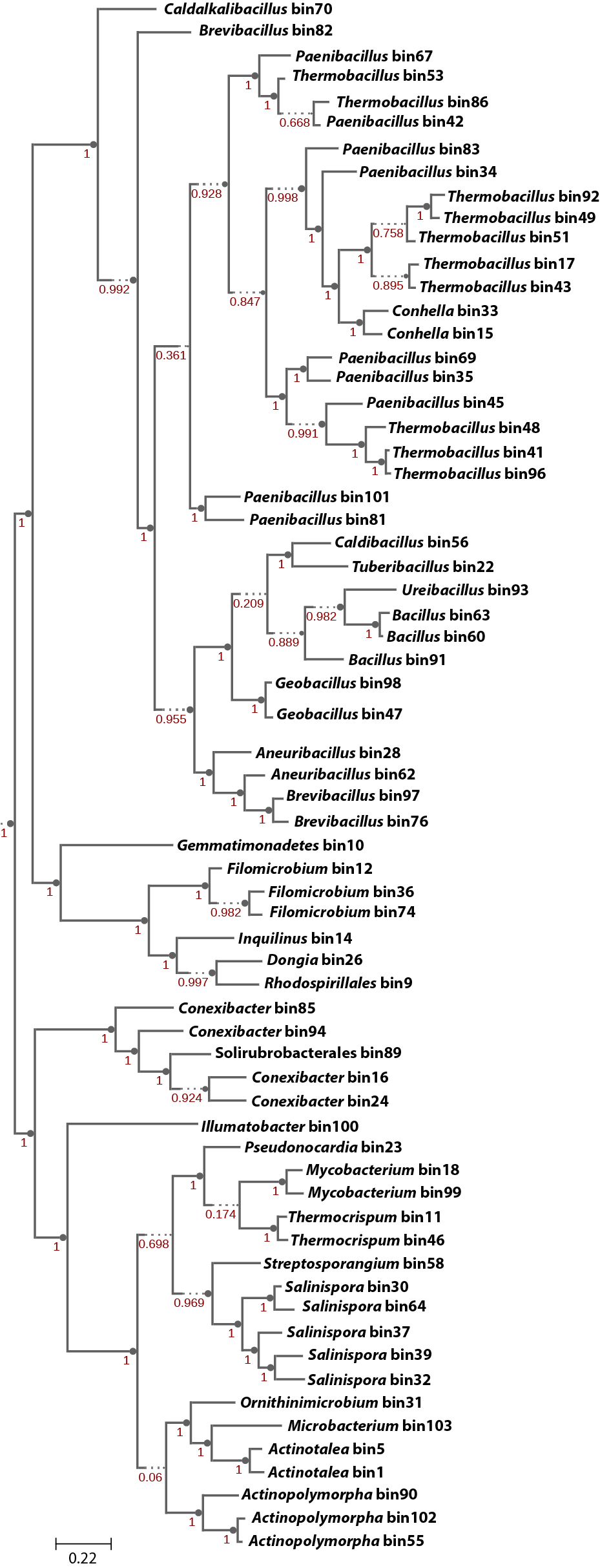


Supplementary Fig. 2. Phylogenetic tree reconstructed based on 49 core universal genes defined by Clusters of Orthologous Groups (COGs) using maximum likelihood. The tree was reconstructed on KBase.


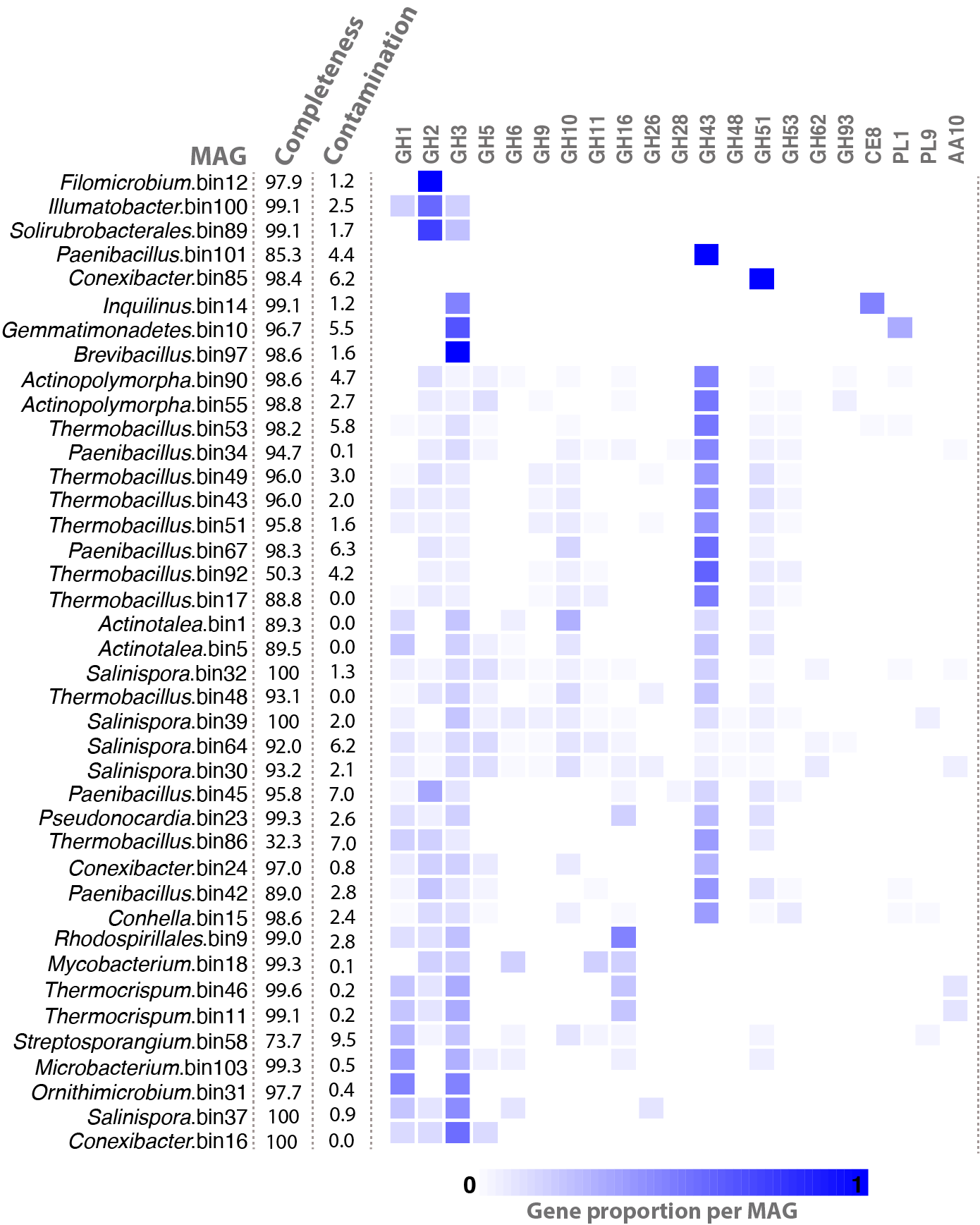


Supplementary Fig. 3. Gene proportion per MAG for selected GHs. MAGs with a completeness above 30% and contamination level lower than 10% are included in the figure.

Supplementary Fig. 4. A) Ordination biplot depicting the trajectory of metatranscriptomes for the adapted communities growing on SM and WT sorghum. The ellipses were calculated around barycenters with a confidence level of 0.99 using the *stat_conf_ellipse*  function in ggpubr v.0.2.4 B) Average TPM-normalized transcriptome abundance per MAG over the 14-day incubation.


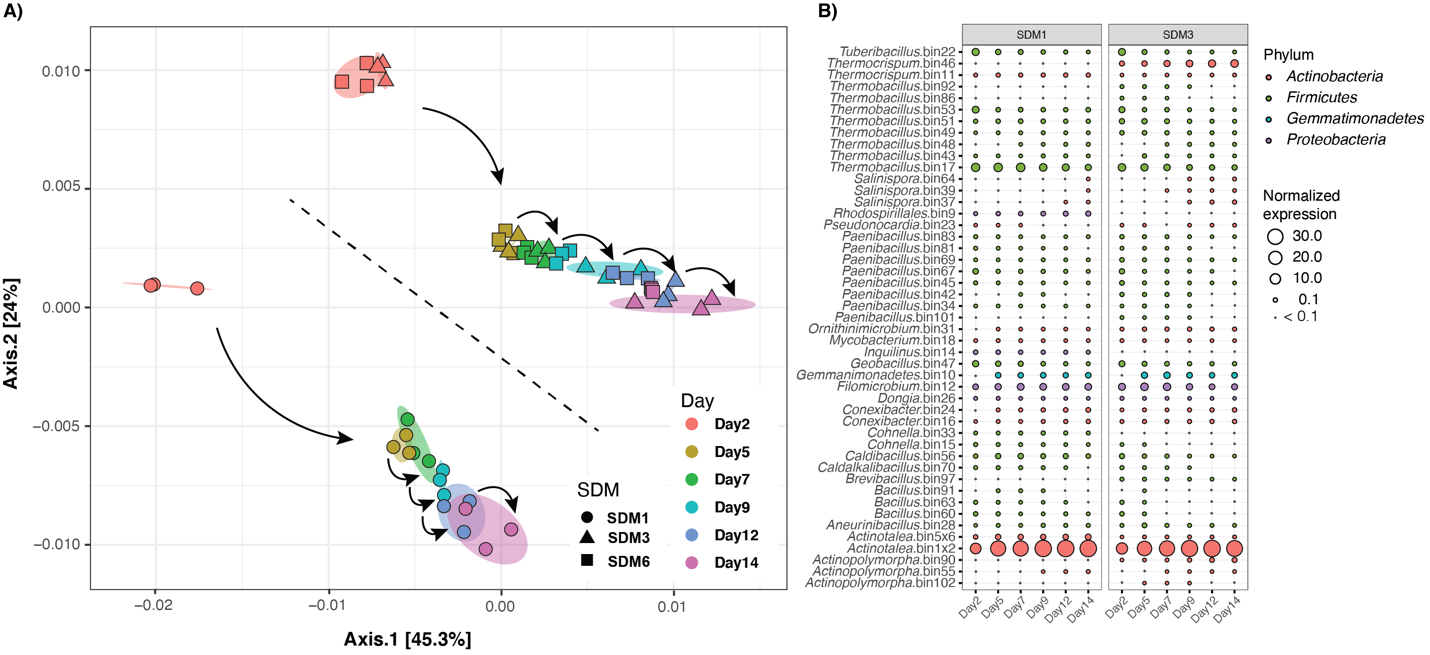

Supplement: Supplementary file 2 — Additional file 1: Supplementary Fig. 1. Residual dry weight biomass after a 56-day incubation. The figure shows that three incubations diverge in terms of biomass degradation. Supplementary Fig. 2. Phylogenetic tree reconstructed based on 49 core universal genes defined by Clusters of Orthologous Groups (COGs) using maximum likelihood. The tree was reconstructed on KBase. Supplementary Fig. 3. Gene proportion per MAG for selected GHs. MAGs with a completeness above 30% and contamination level lower than 10% are included in the figure. Supplementary Fig. 4. A) Ordination biplot depicting the trajectory of metatranscriptomes for the adapted communities growing on SM and WT sorghum. The ellipses were calculated around barycenters with a confidence level of 0.99 using the stat_conf_ellipse function in ggpubr v.0.2.4 B) Average TPM-normalized transcriptome abundance per MAG over the 14-day incubation. [file 40168_2022_1377_MOESM1_ESM.docx]
